# Magnet-controlled capsule endoscopy: the international experience

**DOI:** 10.1016/j.igie.2023.07.010

**Published:** 2023-12-23

**Authors:** Priya Oka, Mohamed G. Shiha, Foong Way David Tai, Ádám Finta, Yu Yuanyuan Karina, Tin Long Marc Wong, James Yun Wong Lau, Laszlo Madacsy, Reena Sidhu, Mark E. McAlindon

**Affiliations:** Academic Unit of Gastroenterology, Sheffield Teaching Hospitals, Sheffield, United Kingdom; Division of Clinical Medicine, School of Medicine and Population Health, University of Sheffield, Sheffield, United Kingdom; Endo-Kapszula Health Centre, Székesfehérvár, Hungary; The Chinese University of Hong Kong, Hong Kong; Endo-Kapszula Health Centre, Székesfehérvár, Hungary; Academic Unit of Gastroenterology, Sheffield Teaching Hospitals, Sheffield, United Kingdom; Division of Clinical Medicine, School of Medicine and Population Health, University of Sheffield, Sheffield, United Kingdom

To the Editor:

We were interested to read the study by Meltzer et al^1^ on magnet-controlled capsule endoscopy (MACE) of the upper GI tract by use of the NaviCam MCCE (AnX Robotica Corp, Plano, Tex, USA), and we believe it will be of interest to compare our experience of the same.

As part of an ongoing multicenter study (NCT04840433), 3 MACE experts assessed the adequacy of upper GI landmark views in 100 patients. Views were adequate in the cardia (88%), fundus (91%), proximal corpus (96%), distal corpus (94%), antrum (94%), and pylorus (91%), consistent with the identification of the same landmarks in >95% of cases by Meltzer et al.^1^ However, in our study, views in the upper, mid, and distal esophagus were adequate in only 68%, 80%, and 60%, respectively. All inadequate views were due to rapid capsule transit. Conversely, both observers in Meltzer et al^1^ viewed the distal esophagus in 97.5% of cases, whereas the Z line was visible in 77.5% and 85% of cases, respectively. The difference might be explained by our stringent requirement of visualization of the whole Z line to consider views of the distal esophagus as adequate. This is important because esophageal disease (reflux, Barrett's esophagus, and cancer) is a major concern in the Western hemisphere, whereas gastric imaging is a priority in China, given the higher prevalence of gastric cancer. Double-headed camera capsules with a high image capture rate provide excellent esophageal imaging,^2^ which suggests that the same could be achieved with capsules developed for gastric imaging.

Patients’ preference for MACE over EGD (79.5%) is consistent with our published findings, where all patients preferred MACE over EGD.^3^ Although MACE is safe, has comparable diagnostic accuracy with that of EGD, and can identify early gastric neoplasia,^4^ our analysis suggested that the cost of MACE was twice that of EGD.^5^ Pricing may become more competitive with more providers and as demand increases. If so, MACE may become a highly desirable alternative to EGD.

## Disclosure

The authors received research material from AnX Robotica Corp to conduct thus study. All authors disclosed no financial relationships.
